# Vascular Immune Crosstalk in COVID-19: RAAS Biomarker Signature Linking Angiotensin II to Respiratory Compromise and Soluble ACE2 to IL-13 and FGF, Revealing Therapeutic Targets

**DOI:** 10.3390/ijms27083579

**Published:** 2026-04-17

**Authors:** Thais Freitas Barreto Fernandes, Itauá Leston Araujo, Pedro Henrique Oliveira Vianna, Adriana Cesar Bonomo, José Henrique Pilotto, Fernanda Heloise Côrtes, Mariza Gonçalves Morgado, Carmem Beatriz Wagner Giacoia-Gripp, Nathalia Beatriz Ramos De Sá, Marcelo Ribeiro-Alves, Maria Pia Diniz Ribeiro, Sandra Wagner Cardoso, Valdilea G. Veloso, Beatriz Grinsztejn, Roberto Magalhães Saraiva, Dalziza Victalina De Almeida

**Affiliations:** 1 Laboratório de Aids e Imunologia Molecular, Instituto Oswaldo Cruz, Fiocruz, Rio de Janeiro 21040-900, Brazil; thaisfbfernandes@gmail.com (T.F.B.F.); fernanda.cortes@fiocruz.br (F.H.C.);; 2 Laboratório de Pesquisa Sobre o Timo, Instituto Oswaldo Cruz, Fiocruz, Rio de Janeiro 21040-900, Brazilphovianna@gmail.com (P.H.O.V.);; 3 Instituto Nacional de Infectologia Evandro Chagas, Fiocruz, Rio de Janeiro 21040-900, Brazilrobertomsaraiva@gmail.com (R.M.S.)

**Keywords:** SARS-CoV-2, cytokines, COVID-19, Ang II, ACE2

## Abstract

COVID-19 perturbs the renin-angiotensin system (RAAS) and inflammatory pathways, shaping disease severity. Soluble ACE2 (sACE2) and angiotensin II (Ang II) are central regulators of vascular and immune homeostasis. We profiled plasma from COVID-19 patients and controls using ELISA, together with 48 cytokine profiling and clinical data. Both sACE2 and Ang II were significantly elevated in patients. Increased Ang II was associated with oxygen supplementation and dyspnea, and negatively correlated with IL-3, whereas sACE2 correlated with IL-13 and FGF. Comorbidities modulated cytokine expression: diabetes mellitus was linked to reduced LIF and MCP-1, hypertension to decreased LIF and increased IP-10, and obesity to elevated IL-12p70. Age correlated with TNF and HGF, and reduced oxygen saturation was associated with lower LIF. These findings reveal that acute COVID-19 disrupts RAAS and amplifies immune dysregulation, with Ang II emerging as a pivotal mediator of respiratory compromise and inflammatory imbalance, underscoring its potential as a biomarker and therapeutic relevance.

## 1. Introduction

Infection caused by severe acute respiratory syndrome coronavirus 2 (SARS-CoV-2) is responsible for the 2019 Coronavirus Disease (COVID-19) [1]. Since the end of the public health emergency designation, COVID-19 has remained an ongoing global threat, with continued transmission and mortality documented in WHO surveillance reports [2,3]. Cumulatively, more than 7.1 million COVID-19 deaths have been officially reported worldwide [3]. In Brazil and the Americas, regional monitoring shows persistent co-circulation of SARS-CoV-2 with other respiratory viruses and episodic increases in activity [4], consistent with national bulletins that track respiratory viruses across seasons [5]; recent InfoGripe analyses indicate that COVID-19 remains the leading cause of deaths among severe acute respiratory infection cases in Brazil [6]. These patterns reinforce the rationale for mechanistic studies that link host pathways to severe outcomes and support targeted interventions.

Symptom severity is influenced by viral mutations and host factors such as age, gender, pre-existing health conditions, and immune responses [7]. Conditions like hypertension, obesity, and diabetes, coupled with advanced age, heighten the risk of severe outcomes [3,4]. Individuals with comorbidities experience heightened inflammation due to the virus’s hyperinflammatory response, resulting in vascular endothelial cell injury, thrombus formation, lung damage, and multi-organ dysfunction [8,9]. Consistently, in a prior study by our group, critically ill COVID-19 patients exhibited significantly higher plasma levels of IL-8, IL-10, TNF-α, interferon-α (IFN-α), IL-1β, IL-17A, IL-23, and IL-6 compared to those with moderate or severe disease [10].

Beyond cytokine dysregulation, accumulating evidence implicates angiotensin-converting enzyme 2 as a key RAAS node in SARS-CoV-2-mediated tissue injury, given its expression on cardiovascular, pulmonary, and renal epithelia [8,11,12]. Viral Spike binding to ACE2 mediates cell entry [10]. Within RAAS, renin converts angiotensinogen to angiotensin I and ACE generate angiotensin II [12], which exerts context dependent vasoactive effects that maintain cardiovascular homeostasis [13]. Physiologically, ACE2 counterbalances this pathway by converting angiotensin II (Ang II) to angiotensin 1–7, activating Mas and promoting vasodilation and natriuresis [14,15,16,17,18]. Emerging data indicate that loss of endothelial ACE2 function during SARS-CoV-2 infection permits des Arg9 bradykinin (DABK) accumulation and B1 receptor overactivation, triggering nitric oxide release, leukocyte trafficking, and blood–brain barrier leakage [19]. Infection driven RAAS modulation likely worsens clinical status in high-risk individuals [16]. Although early RAAS activation may be compensatory, persistent activation promotes inflammation and fibrosis characteristic of cardiovascular and renal disease, thereby compounding COVID-19 pathogenesis and the risk of severe outcomes [20,21,22,23,24]. Importantly, sustained RAAS imbalance and endothelial dysregulation may also contribute to persistent post-acute symptoms consistent with long COVID, reinforcing the need for mechanistic studies and targeted interventions that restore ACE2 Ang 1–7 Mas signaling while restraining downstream B1R Ang II pathways. In addition, recent computational modeling studies have suggested that RAAS peptides such as Ang II and Ang-(1–7) may interact with pathways related to the SARS-CoV-2 main protease (Mpro) [25]. Although still hypothetical, this perspective provides a conceptual link between the RAAS imbalance documented in patients and the activity of a critical viral enzyme, broadening the rationale for investigating how alterations in Ang II and ACE2 influence not only immune and vascular dysfunction but also potentially viral replication.

Understanding how key RAAS components, such as ACE2 and Ang II, interact with the immune response is essential for unraveling their impact on disease progression. Therefore, this study aims to explore the plasmatic levels of ACE2 and Ang II, alongside a detailed panel of cytokines, chemokines, and growth factors, in individuals hospitalized with severe COVID-19. By integrating the acute inflammatory profile with clinical data collected during hospitalization, we aim to identify biomarkers and inflammatory patterns that clarify disease severity and its clinical implications. Our findings provide molecular and clinical evidence to guide future hypothesis-driven evaluations of biomarkers and potential therapeutic targets.

## 2. Results

The study population of COVID-19 participants consisted mainly of middle-aged adults with brown skin color, with a high prevalence of hypertension and diabetes. Regarding comorbidities, the prevalence of obesity or a history of bariatric surgery did not differ between the survivor and non-survivor groups. Likewise, the frequencies of diabetes mellitus, systemic arterial hypertension, chronic obstructive pulmonary disease (COPD), cardiovascular heart failure, chronic kidney disease, thrombosis, dementia, and tuberculosis showed no significant variation between survivors and non-survivors in this cohort. Sociodemographic and clinical characteristics of hospitalized COVID-19 patients are presented in Table 1.

**Figure 1:**
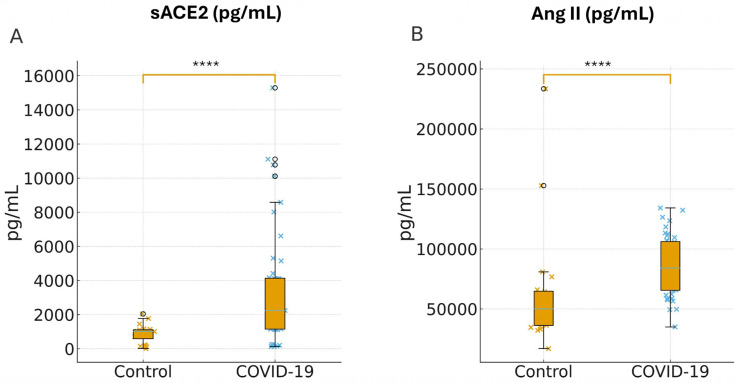
Quantification of soluble angiotensin-converting enzyme 2 (sACE2) and angiotensin II. Panels (**A**,**B**) show plasma sACE2 and Ang II at admission in pg/mL. Dots are individual values, (blue for COVID-19) with slight horizontal jitter; boxes show median and interquartile range; whiskers extend to 1.5× IQR; the bracket indicates the two-sided Mann–Whitney U *p* value. The analysis was performed using GraphPad Prism v 8.0 and the difference between the groups was quantified using the Mann–Whitney test (**** *p*-value < 0.0001).

We compared Ang II and sACE2 concentrations between COVID-19 participants and non-COVID healthy controls. The median age was 57 years (IQR = 18.2) in the COVID-19 group and 37.8 years (IQR = 10.1) in controls, with similar sex distribution between groups (55.8% vs. 45% male, respectively). Plasma levels of sACE2 were significantly higher in COVID-19 patients, with a median of 2246 pg/mL (IQR: 1151–4162), compared with 1021 pg/mL (IQR: 358–1133) in controls (*p * <  0.0001; Figure 1A). Notably, plasma levels of Ang II were also significantly elevated in COVID-19 patients compared with the control group (*p*  <  0.0001), median 84,030 (IQR: 65,590–106,195) pg/mL vs. median 50,060 (IQR: 36,220–64,798) pg/mL, respectively.

**Figure 2:**
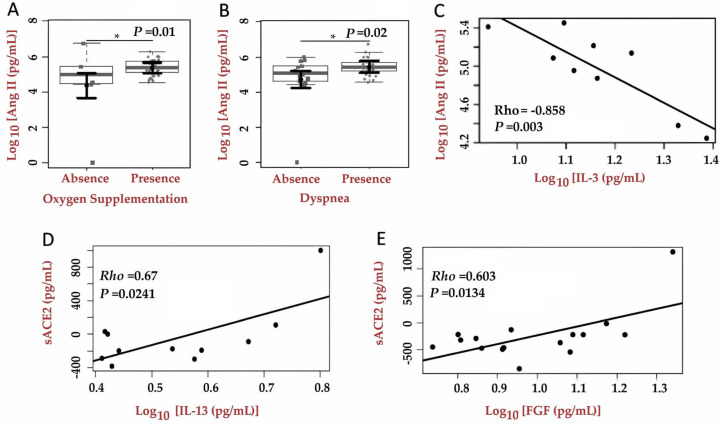
Levels of inflammatory cytokines and correlation analyses between cytokine expression during the acute stage of COVID-19 and the quantification of soluble proteins involved in the RAAS. (**A**) Ang II and oxygen supplementation requirement, (**B**) Ang II levels and presence of dyspnea. (**C**) Correlation between Ang II and Interleukin-3 (IL-3). (**D**) Correlation between ACE2 and Interleukin-13 (IL-13). (**E**) Correlation between ACE2 and fibroblast growth factor (FGF). In (**A**,**B**), the sample distributions of the data are shown in box plots and strip plots in gray. In black, the central circle indicates each group’s expected marginal average effect, estimated from Tobit regression models with multiple fixed effects. Besides groups, the fixed effects in the models included confounding factors sex, age, and days since COVID-19 symptom onset. Black horizontal bars represent the 95% confidence interval for a group’s expected marginal average effects. In (**C**,**D**), Pearson correlation analysis was based on residuals from linear multiple regression models that included sex, age, and days since symptom onset. Rho = Pearson’s correlation coefficient; *p* = significance value associated with the correlation coefficient (* *p*-value < 0.05).

Participants requiring oxygen supplementation exhibited higher Ang II levels, with an estimated mean of 5.37 (95% CI: 5.07–5.67), compared to 4.36 (95% CI: 3.66–5.05) in those not requiring support (*p* = 0.01). Similarly, participants who experienced dyspnea showed elevated Ang II values (estimated mean 5.43; 95% CI: 5.17–5.77) relative to those without dyspnea (estimated mean 4.70; 95% CI: 4.23–5.22; *p* = 0.02) during hospitalization (Figure 2A,B). The analysis of associations with RAAS proteins revealed that a strong negative correlation was observed between Ang II levels and IL-3 cytokine expression (Rho = −0.858; 95%CI: −0.92 to −0.75; *p* = 0.003) (Figure 2C). ACE2 expression showed a positive correlation with IL-13 cytokine (Rho = 0.67; 95%CI: 0.46–0.81; *p* = 0.02) and FGF (Rho = 0.603; 95%CI: 0.37 a 0.76; *p* = 0.01) (Figure 2D,E).

**Figure 3:**
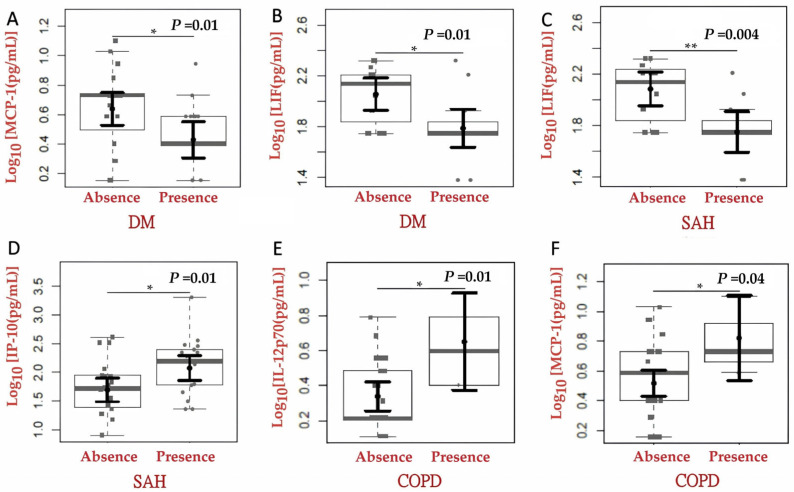
Comparison of inflammatory cytokine, chemokine, and soluble growth factor levels in COVID-19 participants, according to presence or absence of select comorbidities such as diabetes mellitus (DM), systemic arterial hypertension (SAH), and chronic obstructive pulmonary disease (COPD). (**A**) MCP-1 levels in participants with and without DM. (**B**) LIF levels in participants with and without DM. (**C**) LIF levels in participants with and without SAH. (**D**) IP-10 levels in participants with and without SAH. (**E**) IL-12p70 levels in participants with and without COPD. (**F**) MCP-1 levels in participants with and without COPD (* *p*-value < 0.05, ** *p*-value < 0.001).

The results were also analyzed to assess potential associations between cytokine expression and the presence of risk factors such as DM, SAH, COPD, obesity, and age. Study participants with COVID-19 and diabetes had lower plasma concentrations of LIF (1.79; 95% CI: 1.64–1.94) compared to those without diabetes (2.06; 95% CI: 1.93–2.18). Similarly, MCP-1 levels were reduced in participants with DM (0.43; 95% CI: 0.31–0.55) relative to those without the condition (0.64; 95% CI: 0.53–0.75), (*p* = 0.01) (Figure 3A,B). In individuals with SAH, LIF levels were significantly lower (*p* = 0.004), (1.75; 95% CI: 1.59–1.91 vs. 2.08; 95% CI: 1.95–2.21), whereas IP-10 levels were higher (2.07; 95% CI: 1.86–2.29 vs. 1.69; 95% CI: 1.49–1.90) compared to those without hypertension (*p* = 0.01) (Figure 3C,D). Additionally, participants with COPD showed increased quantifications of IL-12p70 (*p* = 0.01) and MCP-1 (*p* = 0.04) (Figure 3E,F).

**Figure 4:**
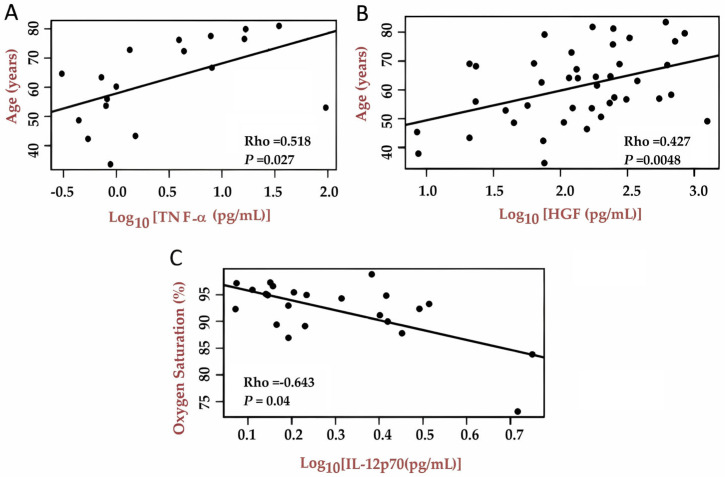
Correlation analyses between the log10-transformed quantification of inflammatory cytokines and participant clinical and sociodemographic data. (**A**) Correlation between participant age (years) and TNF-α levels. (**B**) Correlation between participant age (years) and hepatocyte growth factor (HGF) levels. (**C**) Correlation between oxygen saturation (%) and IL-12p70 levels. Pearson correlation analysis based on residuals from linear multiple regression models that include sex, age, and days since symptom set. Rho = Pearson’s correlation coefficient; *p* = significance value associated with the correlation coefficient.

We found positive correlations between cytokines TNF (Rho = 0.518; *p* = 0.02) and HGF (Rho = 0.427; *p* = 0.0048) plasma levels with age of the COVID-19 participants (Figure 4A,B). We also identified a negative correlation between cytokine IL-12p70 plasma levels and O_2_ saturation during hospitalization (Rho = −0.643; *p* = 0.04) (Figure 4C).

**Figure 5:**
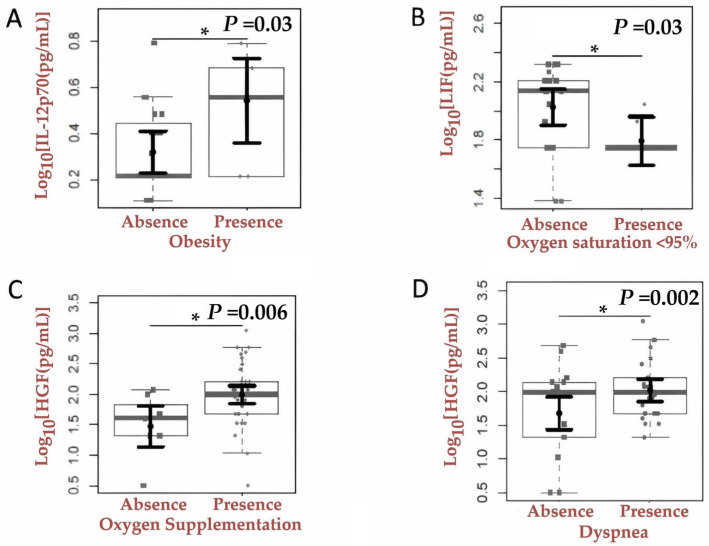
Comparison of inflammatory cytokine, chemokine, and soluble growth factor levels among groups stratified by obesity (present: n = 10; absent: n = 33), oxygen saturation < 95%, need for oxygen supplementation, and presence of dyspnea. (**A**) IL-12p70 levels in participants with and without obesity. (**B**) LIF levels in participants with and without oxygen saturation < 95%. (**C**) HGF levels in participants with and without the need for oxygen supplementation. (**D**) HGF levels in participants with and without dyspnea. The sample distributions of the data are shown in box plots and strip plots in gray. In black, the central circle indicates each group’s expected marginal average effect, estimated from Tobit regression models with multiple fixed effects. Besides groups, the fixed effects in the models included confounding factors such as sex, age, and days since COVID-19 symptom onset. * *p*-value < 0.05.

Furthermore, participants with COVID-19 and obesity exhibited higher IL-12p70 levels (0.54; 95% CI: 0.36–0.73) compared to the non-overweight group (0.32; 95% CI: 0.23–0.41; *p* = 0.03) (Figure 5A). We observed lower plasma levels of LIF in participants with O_2_ saturation below 95% (1.79; 95% CI: 1.63–1.96) compared to those with normal saturation (2.03; 95% CI: 1.90–2.15; *p* = 0.03). In contrast, HGF levels were slightly reduced in participants with low O_2_ saturation (1.86; 95% CI: 1.61–2.10) compared to those without desaturation (1.94; 95% CI: 1.76–2.12). Additionally, higher HGF concentrations were observed in participants who required oxygen supplementation (*p* = 0.006) and in those presenting with dyspnea at admission (*p* = 0.02) (Figure 5B–D). Additional associations involving cytokines outside the direct scope of the RAAS/COVID-19 were not reported in this manuscript.

## 3. Discussion

Numerous studies have investigated the involvement of the RAAS in blood pressure regulation and recently in the pathogenesis of COVID-19 [26]. One of the hypotheses investigated is whether alterations in the RAAS may influence the severity of COVID-19 [27]. In the analysis of soluble proteins ACE2 and Ang II, an increase was observed in the COVID-19 groups compared to the healthy control group. Our findings are consistent with previous reports showing elevated sACE2 in hospitalized individuals, likely reflecting enhanced ectodomain shedding after SARS-CoV-2 engagement of its entry receptor, with concomitant reduction of membrane ACE2 [28]. Such a shift is expected to weaken the ACE2/Ang-(1–7)/MAS1 counter-regulatory axis and favor the ACE/Ang II/AT1R pathway, thereby promoting endothelial dysfunction and inflammation.

Consistent with this model, Ang II was higher in participants who required oxygen supplementation and in those with dyspnea at admission, suggesting a contribution of RAAS activation to respiratory compromise. Although some studies report higher ACE2 expression in younger individuals [29], our cohort had a mean age of 57 years and did not include pediatric cases. In the comparative dataset, however, samples from 21 children (1–18 years), 26 adults (20–60 years), and 29 older individuals (≥60 years) were analyzed, indicating that our findings cannot address age-dependent differences directly. We also observed positive correlations of sACE2 with IL-13 linked to epithelial function and airway remodeling in allergic/asthmatic settings [30] and with FGF, a mediator of injury and fibrotic responses [31], supporting the interpretation that sACE2 may track epithelial injury and tissue-repair programs during acute infection.

Regarding comorbidities, participants with diabetes showed reduced MCP-1 and LIF levels. Although MCP-1 is generally elevated in severe COVID-19, its reduction in diabetes may indicate impaired monocyte-chemotactic signaling or treatment-related effects, rather than a protective role, particularly in the absence of longitudinal data [32]. LIF, a member of the IL-6 family with cytoprotective/regenerative properties, was decreased, which could indicate impaired reparative capacity and potential vulnerability to chronic inflammatory injury [33]. In participants with systemic arterial hypertension, IP-10/CXCL10 was higher, which is an interferon-inducible chemokine repeatedly associated with severe disease and cytokine-storm phenotypes [34], and LIF was lower, together suggesting a more inflammatory and less reparative milieu [35]. Additionally, vitamin D status may shape this comorbidity linked patterns, as acute COVID-19 cohorts have shown lower 25(OH) D_3_ than non-COVID controls and exploratory calcitriol use coincided with IL 6 reductions, raising the hypothesis that vitamin D insufficiency could further bias the RAAS cytokine milieu and warranting longitudinal evaluation alongside 25(OH) D_3_, sACE2, Ang II, and cytokines [36].

According to Coelho et al. (2025), IL-12p70 is one of the inflammatory cytokines found at elevated levels in individuals with COVID-19, suggesting a heightened inflammatory immune response during viral infection [37]. In our study, individuals with COPD also exhibited significantly increased levels of the proinflammatory cytokines IL-12p70 and MCP-1 compared to those without COPD, reinforcing the presence of an exacerbated inflammatory profile in this group. These findings indicate that both COVID-19 and underlying comorbidities such as COPD contribute to a more pronounced proinflammatory immune state. We also observed a negative correlation between higher levels of IL-12p70 and lower O_2_ saturation, and with participants with obesity, indicating the important inflammatory role of the cytokine in the study individuals. In fact, this cytokine has already been associated with worse prognoses in severe COVID-19 patients [38].

The association between age and increases in HGF and TNF levels suggests a progression of the clinical picture as age advances, highlighting why age is considered a significant risk factor in COVID-19. HGF levels were also elevated in participants presenting dyspnea and requiring oxygen supplementation, indicating a potential role for this growth factor in the respiratory complications of the disease. These increased levels suggest ongoing lung tissue injury, as HGF is known to be involved in modulating the immune response by limiting local inflammation, minimizing tissue damage, and promoting tissue repair [39]. It is important to note that cytokine measurements were performed only in plasma samples from COVID-19 participants; given the modest cohort size, the findings should be interpreted as exploratory. To maximize robustness under these constraints, we applied rigorous statistics, including pre-specified group comparisons, appropriate nonparametric tests for skewed distributions, multivariable adjustment for age, sex, BMI, time from symptom onset, comorbidities, and treatments, and we excluded individuals on ACE inhibitors or ARBs to avoid interference with RAAS pathways. Prospective validation in larger, longitudinal cohorts with protocolized sampling is warranted. In plasma collected a mean of 9 days after symptom onset, Ang II and sACE2 were elevated and strongly associated with respiratory compromise and cytokine shifts, a robust and clinically meaningful signal. Crucially, these systemic signatures map directly onto the endothelial mechanism demonstrated in the referenced blood–brain barrier model, where SARS-CoV-2 curtailed membrane ACE2 activity, blunted Ang II to Ang-(1–7) conversion, and unleashed DABK–B1R–driven nitric-oxide production, permeability, and leukocyte and viral transmigration [14]. This congruence strengthens causal inference beyond correlation, positioning the findings as the mechanistic scaffold that explains our circulating biomarker pattern and its respiratory consequences. The increase in circulating sACE2 is most often attributed to ectodomain shedding mediated by ADAM17 following SARS-CoV-2 Spike engagement [40,41]. Although we did not measure ADAM17 activity in this study, in previous work from our group analyzing the same cohort, we observed downregulation of ACE2 and TMPRSS2 expression in severe cases and in individuals with diabetes, accompanied by suppression of ACE2-regulatory microRNAs, including miR-200c, let-7b, and miR-122 [27]. These findings suggest that, in addition to proteolytic shedding, transcriptional and post-transcriptional mechanisms may also contribute to the modulation of ACE2 and to the pool of circulating sACE2 detected in plasma. Consistently, low tissue ACE2 expression in more severe subgroups together with increased circulating sACE2 and Ang II indicates operational loss of ACE2 and RAAS imbalance, tightly linking endothelial dysfunction to hypoxemia and immune dysregulation.

Collectively, these results are decisive and actionable, elevating Ang II and sACE2 as high-value biomarkers and reinforcing, with strong translational relevance, therapeutic strategies that restore the ACE2 Ang-(1–7) axis or attenuate downstream B1R and Ang II signaling.

## 4. Materials and Methods

This prospective, non-randomized study included 43 hospitalized individuals with a confirmed diagnosis of COVID-19 who were admitted to the COVID-19 Center of the Evandro Chagas National Institute of Infectious Diseases (INI-Fiocruz) in Rio de Janeiro, Brazil (RECOVER-SUS study-NCT04807699). Plasma samples from these participants were collected on the first day of hospitalization between June 2020 and December 2021. Additionally, blood samples from 20 healthy individuals collected before the COVID-19 pandemic were used as controls. For all participants, ten milliliters of venous blood were drawn using BD Vacutainer^®^ tubes, Becton, Dickinson and Company, Franklin Lakes, NJ, USA, and demographic and clinical data were recorded at study entry. To assess biomarker expression, ACE2 and Ang II ELISA quantification were performed for comparison with controls, and a cytokine panel was used to examine the expression of 48 biomarkers, including cytokines, chemokines, and growth factors, in plasma samples from COVID-19 patients during the acute SARS-CoV-2 infection. The study was approved by the local Ethics Committee and conducted in accordance with the Brazilian National Committee for Research Ethics and Resolution 466/2012 of the National Health Council, of the Ministry of Health, with all participants providing written informed consent. Eligibility criteria required written informed consent, age 18 years or older, absence of chronic coinfections, not being pregnant, and no current use of medications that could confound RAAS or inflammatory readouts, specifically systemic corticosteroids and renin–angiotensin–aldosterone system inhibitors.

### 4.1. Clinical and Symptomatic Data

At admission, participants provided sociodemographic information including age, sex, and race. Data were also collected on the presence of comorbidities such as diabetes mellitus (DM), systemic arterial hypertension (SAH), chronic obstructive pulmonary disease (COPD), heart failure, chronic kidney disease, thrombosis, dementia, tuberculosis, and obesity. Use of medications including common cardiometabolic agents such as metformin and statins was ascertained at admission and incorporated into covariate review; however, limited detail on dosing and duration precluded definitive adjustment and is noted as a study limitation. In addition, clinical data related to symptoms and hospitalization were collected, including fever, cough, chest pain, rhinorrhea, dyspnea, odynophagia, anosmia, dysgeusia, diarrhea, abdominal pain, nausea, headache, myalgia, oxygen saturation below 95 percent, and the need for oxygen supplementation at the first hospital admission. The mean time from symptom onset to hospitalization was 9 days mean of 8.6 days, standard deviation ± 3.9.

### 4.2. Quantification of Soluble ACE2 and Angiotensin II Proteins by ELISA Technique

Plasma concentrations of angiotensin II (Ang II) and soluble ACE2 (sACE2) were quantified using commercial ELISA kits (Human Angiotensin ELISA Kit-ID UniProt (Humano, San Diego, CA, USA) P01019; Human ACE2 ELISA Kit, ID UniProt (Humano) Q9BYF1; Invitrogen, Carlsbad, CA, USA), following the manufacturer’s protocols. The detection limits were 1.22 ng/mL for Ang II and 0.025 ng/mL for sACE2. Readings were conducted at 450 nm on a plate reader. Optical density (OD) values were converted to pg/mL based on the standard curve of each assay. The resulting concentrations were adjusted for the dilution factor to obtain undiluted values, which were then analyzed using GraphPad Prism v.8.

### 4.3. Quantification Panel of Proinflammatory Cytokines, Chemokines, and Growth Factors by Luminex

Cytokines were measured in undiluted/in a final 25-fold dilution in serum samples. All serum samples were previously stored at −70 °C before analysis. The cytokine panel included the proinflammatory, hematopoietic, regulatory, adaptive, and/or growth-related cytokines: interleukin (IL)-1β, IL-1Rα, IL-1α, IL-2, IL-4, IL-5, IL-6, IL-7, IL-8, IL-9, IL-10, IL-12p70, IL-12p40, IL-16, IL-18, IL-1α, IL-2Rα, IL-3, IL-13, IL-15, IL-17, basic fibroblast growth factor (FGF), cutaneous T-cell attracting chemokine (CTACK), Eotaxin, granulocyte colony-stimulating factor (G-CSF), granulocyte macrophage colony-stimulating factor (GM–CSF), growth regulated (GRO)-α, HGF, interferon (IFN)-γ, IFN-α2, IP-10, leukemia inhibitory factor (LIF), monocyte chemotactic protein (MCP)-1, MCP-3, macrophage colony-stimulating factor (M-CSF), monokine induced by gamma-Interferon (MIG), macrophage migration inhibitory factor (MIF), macrophage inflammatory protein (MIP)–1α, MIP-1β, nerve growth factor (NGF)-β, platelet-derived growth factor (PDGF)-ββ, regulated upon activation normal T cell expressed and presumably secreted (RANTES), stem cell factor (SCF), stem Cell Growth Factor (SCGF)-β, stromal-derived factor (SDF)-1α, TNF, TNF-β, TNF-related apoptosis inducing ligand (TRAIL), and vascular endothelial growth factor (VEGF) measured using Bio-Plex Pro Human Cytokine kit (Screening Panel 48-Plex#12007283, Bio-Rad Laboratories, Hercules, CA, USA) on the MAGPIX Multiplex Reader and the Bio-Plex^®^ 200 System (Bio-Rad) according to the manufacturer’s instructions. Data processing was performed using the software provided by the manufacturer (Bio-Rad Laboratories, Hercules, CA, USA). Recombinant cytokines were used to establish standard curves and the sensitivity of the assay. Results were expressed as Median Fluorescence Intensity (MFI). The MFI of the last point of each standard curve was used to determine the detection limit of each cytokine. Optical density values were converted to pg/mL using the assay’s standard curve, and values were analyzed using GraphPad Prism v.8.

### 4.4. Statistical Analysis

Descriptive analyses were conducted according to patient outcomes (hospital discharge vs. death). For numerical continuous variables, results are presented as median and interquartile range (IQR). Group comparisons were performed using the Mann–Whitney U test. Categorical variables are summarized as absolute and relative frequencies (%) and compared using the chi-squared test. To evaluate differences in cytokine levels according to the presence or absence of comorbidities, cytokine concentrations were log_10_-transformed to address skewed distributions. Tobit regression models were used to estimate mean differences in log10-transformed cytokine levels, adjusting for sex, age, and days since onset of first symptoms. This approach accounts for potential censoring due to assay detection limits. Marginal expected mean values and contrasts were estimated using the ‘emmeans’ package to obtain adjusted comparisons on the original scale. Pearson correlation coefficients were computed to assess pairwise associations among cytokines. Correlations were based on residuals from linear multiple regression models that included sex, age, and days since symptom onset as covariates, thus accounting for potential confounding effects and correlation strengths were categorized as weak (0.3–0.4), moderate (>0.4–<0.7), and strong (≥0.7–1). All statistical analyses were performed in R version 4.1.2. The following packages were used: ‘AER’ (for Tobit models), ‘lme4’ (for linear modeling), and ‘emmeans’ (for estimated marginal means and contrasts).

## 5. Conclusions

Our study provides important insights into how RAAS dysregulation and inflammatory responses contribute to COVID-19 severity. The significant increases in sACE2 and Ang II were notably associated with respiratory complications, including oxygen supplementation and dyspnea, reinforcing their potential as biomarkers of disease progression. Comorbidities such as DM, SAH, and COPD differentially influenced cytokine expression, underscoring their impact on immune dysregulation. The correlations of age with TNF and HGF highlight why older individuals face worse outcomes. Elevated IL-12p70 in participants with obesity, together with its association with lower oxygenation, suggests a potential role in inflammation-mediated tissue damage. The reduction of LIF in patients with DM and SAH may indicate a compromised regenerative response, increasing the risk of long-term complications. Collectively, these findings reveal key pathways underlying COVID-19 severity and underscore the need to advance targeted strategies for high-risk populations. Future research should include a biomarker-guided longitudinal program tracking RAAS and cytokine dynamics alongside endothelial injury markers and define therapeutic windows; a mechanistic preclinical arm using human lung microvascular endothelium, airway organoids, and relevant in vivo models to test short-course angiotensin II as an adjunct vasopressor to determine strategies that restore the ACE2/Ang-(1–7)/MAS1 axis while controlling vasoplegia improve oxygenation, reduce vasopressor exposure, and shorten ICU stay in high-risk subgroups. In sum, this agenda moves from biomarkers to benefit.

## Figures and Tables

**Table 1:** Baseline characteristics of non-COVID controls and COVID-19 participants at admission.

Characteristic	Non-COVID Controls	Acute COVID-19		
		Survivors (n = 32)	Non-Survivors (n = 11)	Total (n = 43)
Age, median (IQR)	37.8 (10.1)	55.6 (15.5)	64.1 (11.1)	57.0 (18.2)
Male sex, n (%)	9 (45%)	19 (59.4%)	5 (45.5%)	24 (55.8%)
Female sex, n (%)	11 (55%)	13 (40.6%)	6 (54.5%)	19 (44.2%)
Hypertension, n (%)	-	15 (46.9%)	5 (45.5%)	20 (46.5%)
Diabetes, n (%)	-	16 (50.0%)	3 (27.3%)	19 (44.2%)
* Obesity, n (%)	-	8 (25.0%)	2 (18.2%)	10 (23.3%)
SAPS3, median (IQR)	-	45 (11)	46 (11)	46 (11)
Oxygen/ventilatory support, n (%)	-	26 (81.2%)	10 (90.9%)	36 (83.7%)

* BMI body mass index. Obesity defined as BMI ≥ 30 kg/m^2^. Continuous variables are shown as percentile (%) or median IQR as appropriate.

## Data Availability

Data will be made available upon request.

## Abbreviations

The following abbreviations are used in this manuscript:

ACE2	Angiotensin-Converting Enzyme 2
Ang II	Angiotensin II
ARB	Angiotensin II Receptor Blocker
BMI	Body Mass Index
COPD	Chronic Obstructive Pulmonary Disease
COVID-19	Coronavirus Disease 2019
CTACK	Cutaneous Tcell Attracting Chemokine
DABK/B1R	Des-Arg9-Bradykinin/B1 receptor
DM	Diabetes Mellitus
FGF	Fibroblast Growth Factor
GCSF	Granulocyte Colony Stimulating Factor
GMCSF	Granulocyte Macrophage Colony Stimulating Factor
GROα	Grow Regulated Oncogene Alpha
HGF	Hepatocyte Growth Factor
IFN	Interferon
IP10	Interferon γ Induced Protein 10
LIF	Leukemia Inhibitory Factor
MCSF	Macrophage Colony Stimulating Factor
MIP	Macrophage Inflammatory Protein
MIF	Macrophage Migration Inhibitory Factor
MFI	Median Fluorescence Intensity
MCP	Monocyte Chemotactic Protein
MIG	Monokine Induced by Gamma Interferon
NGFβ	Nerve Growth Factor Beta
PDGFββ	Platelet-Derived Growth Factor BB
RANTES	Regulated on Activation, Normal T Cell Expressed and Secreted
RAAS	Renin–Angiotensin–Aldosterone System
SCF	Stem Cell Factor
SCGFβ	Stem Cell Growth Factor Beta
SDF1α	Stromal-Derived Factor 1 Alpha
SAH	Systemic Arterial Hypertension
TRAIL	TNF-Related Apoptosis Inducing Ligand
TNF	Tumor Necrosis Factor
VEGF	Vascular Endothelial Growth Factor

## References

[1] Batah S.S., Fabro A.T. (2021). Pulmonary pathology of ARDS in COVID-19: A pathological review for clinicians. Respir. Med..

[2] World Health Organization WHO COVID-19 Dashboard: Summary. https://data.who.int/dashboards/covid19/summary.

[3] World Health Organization COVID-19 Deaths—WHO COVID-19 Dashboard. https://data.who.int/dashboards/covid19/deaths.

[4] Pan American Health Organization Regional Update: Influenza and Other Respiratory Viruses—Epidemiological Week 37, 19 September 2025. https://www.paho.org/en/influenza-situation-report.

[5] Brazil Ministry of Health (2025). Cenário epidemiológico da COVID-19, influenza e outros vírus respiratórios no Brasil: Semana Epidemiológica 1 a SE 52 de 2024. Bol. Epidemiológico.

[6] Fundação Oswaldo Cruz Fiocruz InfoGripe: COVID-19 Is Responsible for the Majority of Deaths among SARI Cases. 2 October 2025. https://agencia.fiocruz.br/infogripe-covid-19-e-responsavel-pela-maioria-dos-obitos-por-srag.

[7] Queiroz M.A.F., Neves P.F.M.D., Lima S.S., Lopes J.D.C., Torres M.K.D.S., Vallinoto I.M.V.C., Bichara C.D.A., Santos E.F.D., de Brito M.T.F.M., da Silva A.L.S. (2022). Cytokine Profiles Associated With Acute COVID-19 and Long COVID-19 Syndrome. Front. Cell. Infect. Microbiol..

[8] Azevedo R.B., Botelho B.G., de Hollanda J.V.G., Ferreira L.V.L., de Andrade L.Z.J., Oei S.S.M.L., Mello T.D.S., Muxfeldt E.S. (2021). Covid-19 and the cardiovascular system: A comprehensive review. J. Hum. Hypertens..

[9] Guzik T.J., Mohiddin S.A., Dimarco A., Patel V., Savvatis K., Marelli-Berg F.M., Madhur M.S., Tomaszewski M., Maffia P., D’acquisto F. (2020). COVID-19 and the cardiovascular system: Implications for risk assessment, diagnosis, and treatment options. Cardiovasc. Res..

[10] De Sá N.B.R., Macieira K.V., Coelho M.R.I., Goulart M.N., Ribeiro-Alves M., Rosadas L.A.d.S., Geraldo K.M., Ribeiro M.P.D., Cardoso S.W., Grinsztejn B. (2025). COVID-19 and HIV: Clinical Outcomes and Inflammatory Markers in a Cohort from a Reference Hospital in Rio de Janeiro, Brazil. Viruses.

[11] Pinto B.G.G., Oliveira A.E.R., Singh Y., Jimenez L., A Gonçalves A.N., Ogava R.L.T., Creighton R., Peron J.P.S., I Nakaya H. (2020). ACE2 Expression is Increased in the Lungs of Patients with Comorbidities Associated with Severe COVID-19. J. Infect. Dis..

[12] South A.M., Brady T.M., Flynn J.T. (2020). ACE2 (Angiotensin-Converting Enzyme 2), COVID-19, and ACE Inhibitor and Ang II (Angiotensin II) Receptor Blocker Use during the Pandemic: The Pediatric Perspective. Hypertension.

[13] Ramos S.G., Rattis B.A.D.C., Ottaviani G., Celes M.R.N., Dias E.P. (2021). ACE2 Down-Regulation May Act as a Transient Molecular Disease Causing RAAS Dysregulation and Tissue Damage in the Microcirculatory Environment Among COVID-19 Patients. Am. J. Pathol..

[14] Cousin V.L., Giraud R., Bendjelid K. (2021). Pathophysiology of COVID-19: Everywhere You Look You Will See ACE2. Front. Med..

[15] Ferreira S.R.G., Zanella M.T. (2000). Sistema renina-angiotensina-aldosterona e nefropatia diabética. Rev. Bras. Hipertens..

[16] Elshafei A., Khidr E.G., El-Husseiny A.A., Gomaa M.H. (2021). RAAS, ACE2 and COVID-19: A mechanistic review. Saudi J. Biol. Sci..

[17] Osman I.O., Melenotte C., Brouqui P., Million M., Lagier J.-C., Parola P., Stein A., La Scola B., Meddeb L., Mege J.-L. (2021). Expression of ACE2, Soluble ACE2, Angiotensin I, Angiotensin II and Angiotensin-(1-7) Is Modulated in COVID-19 Patients. Front. Immunol..

[18] Wang C.-W., Chuang H.-C., Tan T.-H. (2023). ACE2 in chronic disease and COVID-19: Gene regulation and post-translational modification. J. Biomed. Sci..

[19] Coelho S.V.A., e Souza G.L., Bezerra B.B., Lima L.R., Correa I.A., de Almeida D.V., da Silva-Aguiar R.P., Pinheiro A.A.S., Sirois P., Caruso-Neves C. (2025). SARS-Cov-2 Replication in a Blood-Brain Barrier Model Established with Human Brain Microvascular Endothelial Cells Induces Permeability and Disables ACE2-Dependent Regulation of Bradykinin B1 Receptor. Int. J. Mol. Sci..

[20] Te Riet L., van Esch J.H., Roks A.J., van den Meiracker A.H., Danser A.J. (2015). Hypertension: Renin-Angiotensin-Aldosterone System Alterations. Circ. Res..

[21] Silva M.J.A., Ribeiro L.R., Gouveia M.I.M., Marcelino B.d.R., dos Santos C.S., Lima K.V.B., Lima L.N.G.C. (2023). Hyperinflammatory Response in COVID-19: A Systematic Review. Viruses.

[22] Lesgards J.F., Cerdan D., Perronne C. (2025). Do Long COVID and COVID Vaccine Side Effects Share Pathophysiological Picture and Biochemical Pathways?. Int. J. Mol. Sci..

[23] Augustine R., Abhilash S., Nayeem A., Salam S.A., Augustine P., Dan P., Maureira P., Mraiche F., Gentile C., Hansbro P.M. (2022). Increased complications of COVID-19 in people with cardiovascular disease: Role of the renin–angiotensin-aldosterone system (RAAS) dysregulation. Chem. Biol. Interact..

[24] Alexandre J., Cracowski J.-L., Richard V., Bouhanick B. (2020). Renin-angiotensin-aldosterone system and COVID-19 infection. Ann. Endocrinol..

[25] De Souza A.S., de Souza R.F., Guzzo C.R. (2022). Quantitative Structure–Activity Relationships, Molecular Docking and Molecular Dynamics Simulations Reveal Drug Repurposing Candidates as Potent SARS-CoV-2 Main Protease Inhibitors. J. Biomol. Struct. Dyn..

[26] Lundström A., Ziegler L., Havervall S., Rudberg A., von Meijenfeldt F., Lisman T., Mackman N., Sandén P., Thålin C. (2021). Soluble angiotensin-converting enzyme 2 is transiently elevated in COVID-19 and correlates with specific inflammatory and endothelial markers. J. Med. Virol..

[27] Fernandes T.F.B., Pilotto J.H., Cezar P.A., Côrtes F.H., Morgado M.G., Giacoia-Gripp C.B.W., De Sá N.B.R., Cazote A.D.S., Neves A.F., Quintana M.D.S.B. (2025). Modulation of RAAS receptors and miRNAs in COVID-19: Implications for disease severity, immune response, and potential therapeutic targets. BMC Infect. Dis..

[28] Sen R., Sengupta D., Mukherjee A. (2022). Mechanical dependency of the SARS-CoV-2 virus and the renin-angiotensin-aldosterone (RAAS) axis: A possible new threat. Environ. Sci. Pollut. Res..

[29] Silva M.G., Falcoff N.L., Corradi G.R., Di Camillo N., Seguel R.F., Tabaj G.C., Guman G.R., de Matteo E., Nuñez M., Gironacci M.M. (2023). Effect of age on human ACE2 and ACE2-expressing alveolar type II cells levels. Pediatr. Res..

[30] Peebles R. (2022). IL-13 Protects against SARS-CoV-2?. Am. J. Respir. Cell Mol. Biol..

[31] Karadeniz H., Güler A.A., Özger H.S., Yıldız P.A., Erbaş G., Bozdayı G., Bulut T.D., Gülbahar Ö., Yapar D., Küçük H. (2022). The Prognostic Value of Lung Injury and Fibrosis Markers, KL-6, TGF-β1, FGF-2 in COVID-19 Patients. Biomark. Insights.

[32] Cancello R., Henegar C., Viguerie N., Taleb S., Poitou C., Rouault C., Coupaye M., Pelloux V., Hugol D., Bouillot J.-L. (2005). Reduction of Macrophage Infiltration and Chemoattractant Gene Expression Changes in White Adipose Tissue of Morbidly Obese Subjects after Surgery-Induced Weight Loss. Diabetes.

[33] Magno A.L., Herat L.Y., Carnagarin R., Schlaich M.P., Matthews V.B. (2019). Current knowledge of IL-6 cytokine family members in acute and chronic kidney disease. Biomedicines.

[34] Han E., Youn S., Kwon K.T., Kim S.C., Jo H.-Y., Jung I. (2024). Disease progression associated cytokines in COVID-19 patients with deteriorating and recovering health conditions. Sci. Rep..

[35] Metcalfe S.M. (2020). COVID-19 lockdown: De-risking exit by protecting the lung with leukaemia inhibitory factor (LIF). Med. Drug Discov..

[36] Gallelli L., Mannino G.C., Luciani F., de Sire A., Mancuso E., Gangemi P., Cosco L., Monea G., Averta C., Minchella P. (2021). Níveis séricos de vitamina D em indivíduos testados para SARS-CoV-2: Quais são as diferenças entre pacientes com COVID-19 agudo, curado e negativo? Um estudo multicêntrico de prática real. Nutrientes.

[37] Coelho M.M., Moreira F.C., Zuccherato L.W., Ventura L.H.D.A., Camatta G.C., Starling-Soares B., Torres L., Durso D.F., Sato H.I., da Costa M.S. (2025). Living in endemic area for infectious diseases is associated to differences in immunosenescence and inflammatory signatures. Front. Immunol..

[38] Moll-Bernardes R., de Sousa A.S., Macedo A.V.S., Lopes R.D., Vera N., Maia L.C.R., Feldman A., Arruda G.D.A.S., Castro M.J.C., Pimentel-Coelho P.M. (2021). IL-10 and IL-12 (P70) Levels Predict the Risk of Covid-19 Progression in Hypertensive Patients: Insights From the BRACE-CORONA Trial. Front. Cardiovasc. Med..

[39] Zaira B., Yulianti T., Levita J. (2023). Correlation between Hepatocyte Growth Factor (HGF) with D-Dimer and Interleukin-6 as Prognostic Markers of Coagulation and Inflammation in Long COVID-19 Survivors. Curr. Issues Mol. Biol..

[40] Zipeto D., Palmeira J.D.F., Argañaraz G.A., Argañaraz E.R. (2020). ACE2/ADAM17/TMPRSS2 interplay may be the main risk factor for COVID-19. Front. Immunol..

[41] Lambert D.W., Yarski M., Warner F.J., Thornhill P., Parkin E.T., Smith A.I., Hooper N.M., Turner A.J. (2005). Tumor Necrosis Factor-α Convertase (ADAM17) Mediates Regulated Ectodomain Shedding of the Severe-acute Respiratory Syndrome-Coronavirus (SARS-CoV) Receptor, Angiotensin-converting Enzyme-2 (ACE2). J. Biol. Chem..

